# A Novel Neutral and Mesophilic β-Glucosidase from Coral Microorganisms for Efficient Preparation of Gentiooligosaccharides

**DOI:** 10.3390/foods10122985

**Published:** 2021-12-03

**Authors:** Hongfei Su, Qi Zhang, Kefu Yu, Chunrong Lu, Zhenlun Xiao, Qinyu Huang, Shuying Wang, Yinghui Wang, Guanghua Wang, Jiayuan Liang

**Affiliations:** 1 Coral Reef Research Center of China, Guangxi Laboratory on the Study of Coral Reefs in the South China Sea, School of Marine Sciences, Guangxi University, Nanning 530004, China; shf2016@gxu.edu.cn (H.S.); zhangqi1780@163.com (Q.Z.); chunrlu@163.com (C.L.); xzlgxu@sina.com (Z.X.); hyhqy172@sina.com (Q.H.); wyh@gxu.edu.cn (Y.W.); wgh@gxu.edu.cn (G.W.); jyliang@gxu.edu.cn (J.L.); 2 Southern Marine Science and Engineering Guangdong Laboratory, Zhuhai 519000, China; 3 School of Resources, Environment and Maters, Guangxi University, Nanning 530004, China; wangshuying1910@163.com

**Keywords:** gentiooligosaccharides, β-glucosidase, coral microorganism, reverse hydrolysis, Transglycosylation

## Abstract

β-glucosidases can produce gentiooligosaccharides that are lucrative and promising for the prebiotic and alternative food industries. However, the commercial production of gentiooligosaccharides using β-glucosidase is challenging, as this process is limited by the need for high thermal energy and increasing demand for the enzyme. Here, a putative β-glucosidase gene, selected from the coral microbial metagenome, was expressed in *Escherichia coli*. Reverse hydrolysis of glucose by Blg163 at pH 7.0 and 40 °C achieved a gentiooligosaccharide yield of 43.02 ± 3.20 g·L^−1^ at a conversion rate of 5.38 ± 0.40%. Transglycosylation of mixed substrates, glucose and cellobiose, by Blg163 consumed 21.6 U/0.5 g glucose/g cellobiose, achieving a gentiooligosaccharide yield of 70.34 ± 2.20 g·L^−1^ at a conversion rate of 15.63%, which is close to the highest yield reported in previous findings. Blg163-mediated synthesis of gentiooligosaccharides is the mildest reaction and the lowest β-glucosidase consumption reported to date.

## 1. Introduction

The major component of gentiooligosaccharides is gentiobiose along with small amounts of gentiotriose and gentiotetraose, which are new functional oligosaccharides consisting of two or more D-glucose linked through β-1,6-glycosidic bonds [1,2]. The structural conformation of certain chemical linkages on gentiooligosaccharides enables them to be resistant to human digestive enzymes. Therefore, gentiooligosaccharides are low in terms of calories and have less chances of causing obesity, high blood lipids and pressure, diabetes, and dental caries [3]. Moreover, gentiooligosaccharides can promote the growth of *Bifidobacteria* and *Lactobacillus* as probiotics, enhance vitamin synthesis, improve metabolism, inhibit the proliferation of harmful bacteria and tumors, and promote host immune response and nutrient absorption [3,4,5]. One of the characteristic features of gentiooligosaccharides is hygroscopicity, which helps in maintaining moisture in food, preventing its aging, and increasing its shelf life [5]. They also improve the sensory properties of food by imparting a bitter but soft and refreshing taste; thus, they are useful as an additive and preservative in the food industry. To date, gentiooligosaccharides have been employed widely in coffee, spices, chocolate, ice cream, baking products, and dairy beverages [6].

Extraction from raw materials and enzymatic conversion are two main methods to prepare gentiooligosaccharides. The extraction method yields gentiooligosaccharides from the roots or stems of gentian, and bitter almond benzene. It can also be purified from the by-products of acid-hydrolyzed starch. However, these methods are complex and inefficient, and are limited by the availability of raw materials, which is difficult to meet the high demands of industrial production. The enzymatic conversion method involves the synthesis of oligosaccharides using glucose or/and cellobiose as the substrate by the reverse hydrolysis or transglycosylation of β-glucosidase. Notably, this conversion method offers the advantages of simple operation, reduced reaction steps, easy separation and purification, and high yields [7]. However, large-scale industrial production of gentiooligosaccharides via enzymatic conversion is restricted by the high demand for the β-glucosidase enzyme.

To date, research on gentiooligosaccharide production by the enzymatic conversion method has mainly concentrated on the reverse hydrolysis of β-glucosidase that requires high levels of glucose as the raw material. β-glucosidase also displays transglycosylation activity, which is capable of synthesis of gentiooligosaccharides via the cleaving of β-1,4-glycosidic bonds of cellobiose and the transfer of substrates to glucose for β-1,6-glycosidic bond formation [8,9,10]. A previous study reported that a crude β-glucosidase produced by *Aspergillus oryzae* achieved a yield of 30.86 g·L^−1^ with cellobiose and glucose as the substrate within 72 h at pH 5 and 55 °C [11]. In other studies, recombinant β-glucosidases expressed in different microorganisms, such as *Escherichia coli* [1,12] *and Pichia pastoris* [13], were used to produce gentiooligosaccharides with transglycosylation activity. In a recent study by Wang et al., a β-glucosidase (bgl1) and a protein disulfide isomerase were co-expressed in the *Pichia pastoris* to synthesize gentiooligosaccharides with reverse hydrolysis, as well as transglycosylation [6]. Few studies have investigated the synthesis of gentiooligosaccharide based on β-glucosidase performed under hypothermal or mesothermal conditions.

β-glucosidases (EC 3.2.1.21) are a typical member of the cellulases that catalyze the hydrolysis of the glycosidic bonds in beta-D-glucosides and various oligosaccharides, including disaccharides, aryl-β-D-glucosides, cyanogenic glucosides, alkyl-β-D-glucosides, and other short-chain oligosaccharides, to release glucose [14]. A number of novel β-glucosidases exhibit β-galactosidase and β-xylosidase activity or reverse hydrolysis activity for the synthesis of alkyl glucosides and gentiooligosaccharides [14,15]. These enzymes are prevalent in plants, animals, including non-cellulolytic organisms, such as humans, and microorganisms, including bacteria, such as *Escherichia coli*, or fungi, such as *Aspergillus niger* and *yeast* [14,16]. There are a large number of microorganisms in coral hosts that possess a full range of cellulase enzymes, including endoglucanases, exoglucanases, and β-glucosidases, which are used to digest the surrounding plankton [17] and zooxanthellae to establish enhanced symbiotic relationships [18,19]. More than ten glycosidases have been found in the commensal *Photobacterium mandapamensis*, which are modestly induced on coral mucus and are antagonistic to the regulation of glucose, galactose mannose, arabinose, and N-acetyl-glucosamine, relating to the defensive capability of the coral host [20]. In our previous research, the number of β-glucosidase-producing strains was more than that of protease-producing strains in corals that live at 28 °C and harbour numerous mesophilic bacteria [21]. These results suggested that considerable amounts of β-glucosidase are ubiquitous in coral symbiotic microorganisms. However, there is no information on exploiting these enzymes from corals and their application in the food industry.

In the present study, a novel gene, *blg163*, was selected from the metagenomic library of coral microorganisms using high-throughput sequencing and functional screening. After successful identification and isolation, cloning and overexpression of this gene was performed to obtain the recombinant β-glucosidase, Blg163. The recombinant protein was then characterized and assessed for its application in the industrial production of gentiooligosaccharides.

## 2. Materials and Methods

### 2.1. Strains, Vectors, and Reagents

pEASY-E1(+) (Transgen, Beijing, China) was used to express the recombinant protein in *Escherichia coli* BL21(DE3) pLysS (Novagen, Darmstadt, German) as the vector. T4 DNA ligase, *E. coli* Trans5α, restriction endonucleases, TransFast^®^ Taq DNA polymerase and isopropyl-β-D-1-thiogalactopyranoside (IPTG) were obtained from Transgen (Beijing, China). *p*-Nitrophenyl-β-D-glucopyranoside (*p*NPG), gentiobiose, and cellobiose were obtained from Sigma-Aldrich (Shanghai, China). N-propanol, ethyl acetate and ammonia solution were obtained from Sangon Biotech (Shanghai, China). The DNA extraction kit was purchased from Mega (Mega Co., Ltd., Guangzhou, China). Other chemical reagents used in this study were all analytically pure unless otherwise requested. Luria-Broth comprised yeast extract (5.0 g·L^−1^), tryptone (10.0 g·L^−1^), and NaCl (10.0 g·L^−1^), and the pH was maintained at 7.0, with or without supplementation with agar (15.0 g·L^−1^).

### 2.2. Coral Sample Collection, Activity Screening, DNA Extraction, and High-Throughput Sequencing

Coral samples collection (10 cm × 10 cm) was performed at the Luhuitou coral reef (109°28 E, 18°13 N), located in the west of Luhuitou Peninsula, which is situated on Sanya Bay of the South China Sea, in September 2017. The surfaces of coral samples were washed gently with sterile seawater and transported immediately to the laboratory off-shore for isolation of β-glucosidase-producing bacteria according to Bruce Eberhart’s protocol [22] and our previous study [21] with minor modifications. Total genomic DNA was extracted from all the strains that were screened using a DNA extraction kit, following the manufacturer’s instructions, with minor modifications. Genomic DNA aliquots from the screened strains were submitted to Majorbio (Shanghai, China) for high-throughput sequencing, and the rest was preserved as a template for amplification.

### 2.3. The Recombinant β-Glucosidase Blg163 Expression and Purification

Based on gene annotation, the β-glucosidase gene *blg163* was selected and amplified using the primer pair of 5′-GCATG*GAATTC*TGGTGACAAAAGACATTAAAGCTCTTATTTCTCAGATGA-3′ (*Eco*R I digestion site underlined) and 5′-CCTCCG*CTCGAG*TTTCACAACAGGATTCTTCTGTACCTCATTTAACT-3′ (*Xho* I digestion site underlined). Then, the PCR product was cloned in the doubly digested pEASY-E1(+) empty vector to generate the recombinant plasmid pEASY-*blg163*, which was transformed into *E. coli* DH5α. The purified recombinant plasmids were subsequently transformed into *E. coli* BL21(DE3) pLysS for target protein expression. The transformed cells carrying pEASY-*blg163* were inoculated in LB medium supplemented with ampicillin (100 µg/mL) at 37 °C. When the OD_600_ of the cultured bacterial cells reached 0.5~0.6, 0.6 mM of IPTG was added for further induction at 22 °C. Then, induced cells were harvested for 16 h, washed twice with phosphate buffered saline (PBS buffer, pH 7.0), and lysed by ultrasonication (120 W, 4 s) on ice for 25 min. The lysate was centrifuged at 8200× *g* and 4 °C for 20 min. The supernatant was loaded onto a Ni^2+^-NTA agarose gel column for enzyme purification. Elution buffer (0.3 M NaCl, 0.2 M imidazole, and 20 mM Tris-HCl, pH 7.9) was used to elute the protein and then was diluted three times with 4 °C deionized double-distilled H_2_O. The purified protein was analyzed using sodium dodecyl sulfate-polyacrylamide gel electrophoresis (SDS-PAGE). Determination of the protein concentration was according to the Bradford method with bovine serum albumin as the standard [23].

### 2.4. Activity Assay of β-Glucosidase

The enzyme activity of the recombinant β-glucosidase was determined using *p*NPG as the substrate according to previous report [24]. In brief, the reaction mixture contained 10 μL of appropriately diluted enzyme, 15 μL of 25 mM *p*NPG, and 175 μL of 0.2 M citrate buffer (pH 7.0). After incubation at 30 °C for 5 min, the reaction was terminated by 0.1 M Na_2_CO_3_. The amount of *p*NP released was determined by measuring the absorption of the reaction mixture at 405 nm by spectrometer (Thermo Fisher Scientific Oy, Ratastie 2, FI-01620 Vantaa, Finland). A sample which was added 100 μL of 1 M Na_2_CO_3_ before the addition of the enzyme was set as control. The amount of enzyme that hydrolyzes 1 μmol of *p*NPG per min under the experimental conditions described above was defined as one unit of β-glucosidase activity (U) [25]. To determine the kinetic parameters of the β-glucosidase, such as *Km* and *Vmax*, *p*NPG, cellobiose, or gentiandisaccharide were prepared in a range of concentrations (0.25–2.5 mM) in citrate buffer (pH 7.0) and were incubated with the enzyme at 30 °C for 5 min [26]. The conditions for starting and stopping the reaction were set as same as mentioned earlier.

### 2.5. Characterization of Purified Blg163

Purified Blg163 was characterized using *p*NPG as the substrate. Synthesis of gentiooligosaccharides was detected using glucose and cellobiose as substrates. The optimal pH for the enzymatic reaction was determined using buffers with different pH from 3.0 to 11.0 at 30 °C (pH 8.0–11.0 with 0.05 M glycine-NaOH buffer, pH 3.0–8.0 with 0.2 M McIlvaine buffer). The optimum temperature for enzymatic reaction was determined to be from 0 °C to 70 °C in a buffer with pH 7.0. The residual activity of the enzyme was measured after incubating the enzyme at different temperatures from 0 °C to 70 °C for 1 h to determine the thermal stability of recombinant Blg163. To investigate the effects of different metal cations on the enzyme activity, 1 mM and 10 mM (final concentration) of Mg^2+^, Li^+^, Na^+^, K^+^, NH_4_^+^, Ca^2+^, Fe^3+^, Ba^2+^, Zn^2+^, Ni^2+^, Cu^2+^, Co^2+^, diamine tetra-acetic acid (EDTA), and sodium dodecyl sulfate (SDS) were added to the reaction system individually. The effect of glucose and ethanol on enzyme activity was investigated by measuring the residual activity at different final concentrations (0%, 5%, 10%, 15%, and 20% glucose; 0%, 5%, 10%, 15%, 20%, 25%, and 30% ethanol). The sample with PBS (pH 7.0) was used as the control. All enzyme activities were determined by three repeated experiments.

### 2.6. Gentiooligosaccharide Production via β-Glucosidase-Mediated Reverse Hydrolysis with Glucose

Gentiooligosaccharide synthesis was initiated in a total volume of 2 mL in an orbital shaker (Tsc 24 × 2.0 mL, Biometra, German) at 150 rpm. To evaluate the effect of temperature and pH on yield of gentiooligosaccharides, enzymatic reactions were conducted at a pH ranging from 5.0 to 8.0 and temperatures ranging from 25 to 45 °C in 50 mM McIlvaine buffer (pH 7.0) dissolving 20% (*w/v*) glucose with 40 U of enzyme. The effect of glucose concentration on the yield of gentiooligosaccharides was studied at the optimal pH and temperature with different glucose concentrations (5–50%) incubated with 40 U of the enzyme. The effect of enzyme concentration on the yield of gentiooligosaccharides with 10, 20, 30, 40, and 100 U/g of glucose or U/0.5 g cellobiose was investigated under optimal conditions.

### 2.7. Gentiooligosaccharide Synthesis Using Glucose and Cellobiose via Transglycosylation

Cellobiose and glucose as the donor and acceptor, respectively, were used to produce gentiooligosaccharides. The gentiooligosaccharide synthesis was carried out in 50 mM citrate buffer (pH 7.0) at 30 °C. The effects of molar ratio of substrate (glucose:cellobiose = 1:2, 1:1, 2:1, and 4:1), substrate concentration (glucose + cellobiose = 5% + 20%, 10% + 20%, 10% + 10%, 20% + 10%, 25% + 12.5%, 30% + 15%, 50% + 25%, and 60% + 30%), and enzyme concentration (3.6, 7.2, 14.4, 21.6, 28.8, and 36 U/g of glucose) on the yield of gentiooligosaccharides were determined under optimal conditions. Samples were taken at different time intervals and immediately boiled for 10 min for high performance liquid chromatography (HPLC) analysis.

### 2.8. Thin-Layer Chromatography (TLC) and High-Performance Liquid Chromatography Analysis of Gentiooligosaccharides Produced by Blg163

The synthesis of gentiooligosaccharides was carried out by transglycosylation reaction under optimal conditions, as described above. The resulting samples were analyzed using thin-layer chromatography (TLC silica gel G25, Merck, Drmstadt, Germany). The mobile phase for the TLC comprised n-propanol, ammonia solution, ethyl acetate, and water (6:3:3:1, *v*/*v*). The spots of the samples were visualized with iodine vapor [27]. For assessment of the yield of gentiooligosaccharides, the resulting samples were filtered through a 0.22 μm syringe filter (Merck Millipore Ltd., Drmstadt, Germany), and 20 μL of filtrate was injected into HPLC at 40 °C by a 1260 Infinity II pump, and exposed to a G7102A evaporative light scattering detector (ELSD) (Agilent Technologies, Inc., Santa Clara, CA, USA) equipped with Spherisorb Amino (NH_2_) column (4.6 mm × 250 mm, 80 Å, 5 µm) (Cosmosil, Kyoto, Japan). The mobile phase for HPLC consisted of 25% MilliQ water and 75% acetonitrile with a flow rate of 1.0 mL/min. The retention time of glucose, cellobiose, gentiooligosaccharides was 10.5 ± 0.2, 16.0 ± 0.2, 19.5 ± 0.2 min, respectively, and the calibration curve concentration ranged from 1.00 to 10.00 mg/mL. The maximal enzyme conversion was defined as 100%, and the relative conversion rate for each reaction was calculated accordingly. All analyses were performed in triplicate.

### 2.9. Bioinformatic Analysis and Nucleotide Sequence Submission

The nucleotide sequence of the β-glucosidase gene, *blg163*, was deposited in GenBank (https://www.ncbi.nlm.nih.gov/genbank/ (accessed date: 6 March 2020)) with accession number (MT797857). The three-dimensional structure of Blg163 was predicted by homology modeling (https://swissmodel.expasy.org/ (accessed date: 28 May 2020)) [24]. DNA and protein statistics were analyzed using the algorithms of the sequence-manipulation suite. The signal peptide and catalytic domain of the protein were predicted by the online SMART tool (http://smart.embl-heidelberg.de/ (accessed date: 6 March 2020)). Multiple amino acid sequences of proteins were aligned using the ENDscript server (http://espript.ibcp.fr/ESPript/ESPript/ (accessed date: 9 April 2021)) [28] and ClustalX.

## 3. Results

### 3.1. Cloning, Expression, and Purification of β-Glucosidase

**Figure 1:**
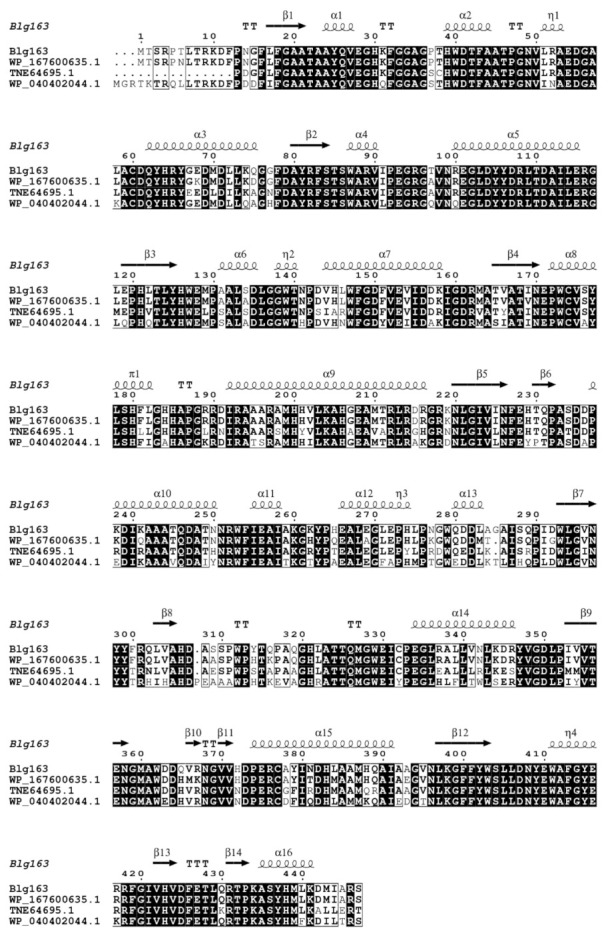
Alignment of amino acid sequence of Blg163 with other β-glucosidases.

Approximately 264 different β-glucosidase-producing strains were obtained from coral, and their metagenome sequencing and annotation were carried out. From the metagenome analysis, a candidate β-glucosidase gene, *blg163*, was identified. This gene was cloned into pEASY-E1(+) and expressed in *Escherichia coli* BL21(DE3) pLysS strain. A complete open reading frame of 1341 bp in the putative β-glucosidase gene shared the highest identity (80%) with a gene without annotation from *Celeribacter ethanolicus* strain TSPH2. This gene encodes a polypeptide with 447 amino acid residues that showed the highest amino acid sequence identities of 95.3%, 94.6%, 83.2%, and 74.4% with putative proteins from the genome of *Rhodobacteraceae bacterium*, *Celeribacter* sp. HF31, *Rhodobacteraceae bacterium*, and *Pacificibacter maritimus*, respectively (Figure 1), which have not so far been characterized. The amino acid residues from Met1 to Ser447 of the Blg163 protein resembled the catalytic domain of the glycosyl hydrolase family 1 (GH1) proteins.

**Figure 2:**
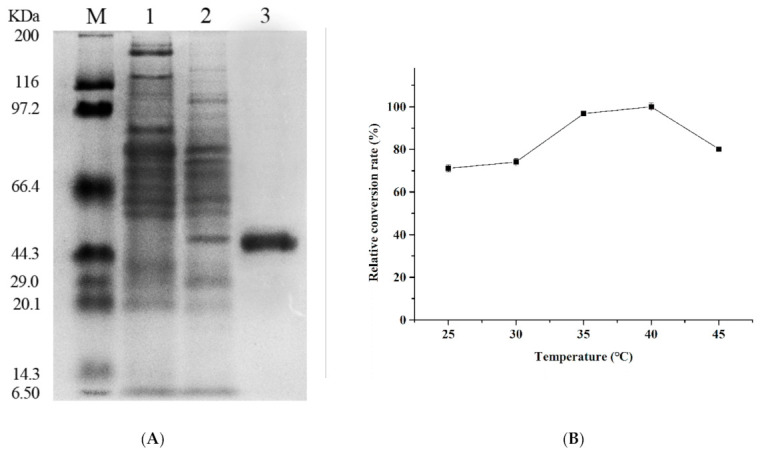
SDS-PAGE analysis of the purified Blg163 (**A**). M protein marker; 1 recombinant *Escherichia coli* BL21(DE3) harboring pEASY-E1(+) induced with IPTG; 2 recombinant *Escherichia coli* BL21(DE3) harboring pEASY-E1(+)—*Blg163* induced with IPTG; 3 purified Blg163. Effects of temperature on gentiooligosaccharide production (**B**). The reactions were launched in 50 mM citrate buffer (pH 7.0) at 25–45 °C with 30% glucose and 15% cellobiose.

β-glucosidase expression was induced by adding IPTG (0.6 mM) to the culture when OD_600 nm_ reached 0.5 at 160 rpm and 22 °C for 16 h. The cell lysate was applied to a Ni^2+^-NTA agarose and washed out with an imidazole gradient of 200–300 mM. The purified protein was subjected to SDS-PAGE, and a single protein band appeared clearly at 54.3 kDa (Figure 2A), which was consistent with the theoretical molecular weight. The eluted fraction was tested for its activity and was demonstrated to be 465 ± 5.6 U·mL^−1^ product generation. Compared to other over-expressed β-glucosidases, such as, the β-glucosidase from *A. niger* AS3.4523, which exhibited high activity (25.88 ± 0.45 U·mL^−1^) [29], Blg163 was successfully expressed in *Escherichia coli* BL21 (DE3) pLysS.

Blg163, β-glucosidase from coral microorganism in this study; *R. b.* NVK46632.1, β-glucosidase from *Rhodobacteraceae bacterium*; *C*. sp. WP_167600635.1, β-glucosidase from *Celeribacter* sp. HF31; R. b. TNE64695.1, β-glucosidase from *Rhodobacteraceae bacterium*; *P. m*.WP_123791906.1, β-glucosidase from *Pacificibacter maritimus*. The similar and conserved amino acids of β-glucosidases were indicated with solid and black boxes, respectively.

### 3.2. Enzymatic Properties of Recombinant β-Glucosidase

Blg163 functioned at an optimal temperature of 30 °C. Moreover, the enzyme maintained its activity up to 35.2% at 10 °C. The enzyme retained >95% residual enzyme activity after incubation at 0–30 °C for 1 h (Figure S1), indicating that it is a cold-active enzyme. Blg163 displayed >90% residual activity between pH 6.0–7.0 and demonstrated the highest activity at pH 7.0, retaining >95% residual enzyme activity after incubation at pH 5.0–8.0 for 1 h (Figure S2), indicating that it is a neutral enzyme. This finding differs from the previously reported β-glucosidases, such as HML0366 (pH 5.0) [11], RmBglu3B [13], and Bgl1 (pH 5.0) [6]. The optimum reaction temperature and pH of Blg163 were confirmed to be 30 °C and 7.0, indicating that this enzyme is a mesophilic and neutral β-galactosidase. Blg163 exhibited >40% of maximum activity with the existence of 5–20% glucose (Figure S3) and retained >50% residual enzyme activity undergoing 15% ethanol incubation (Figure S4), signifying its high tolerance to ethanol and glucose.

The purified recombinant enzyme displayed a peak specific activity of 29.74 ± 2.7 and 28.0 ± 2.3 U·mg^−1^ for *p*NPGlu and cellobiose (Table S1), respectively. The kinetic parameters of Blg163 for *p*NPG, cellobiose, and gentiobiose were measured at the optimum reaction temperature and pH. The *Km* values for *p*NPG, cellobiose, and gentian disaccharide were 0.32, 0.45, and 0.72 mM (Table S1), respectively. These results indicate that Blg163 has a higher substrate affinity for *p*NPGlu than for cellobiose and gentiobiose.

### 3.3. Gentiooligosaccharide Production from Cellobiose and Glucose

Gentiooligosaccharides are widely applied in food processing as they are indigestible [3] and excellent prebiotics [4], can retain moisture, and have a soft, refreshing, bitter taste [6]. It is very important to develop a cost-effective method that achieves mild reaction conditions and simple isolation techniques for preparation of gentiooligosaccharides via enzyme catalysis for the food industry [30]. Some β-glucosidases have been shown to have transglycosylation activity which facilitates the synthesis of gentiooligosaccharides. These enzymatic processes have the advantages of strong specificity, mild reaction conditions, high catalytic efficiency, and easy isolation from the product mixture [31,32]. The reported β-glucosidases, using a high concentration of glucose as the substrate, can transfer free glucose to other sugar substrates by forming β-1,6-glycosidic bonds, thereby synthesizing oligosaccharides [8,33,34].

The effect of glucose concentration on the yield of enzymatic catalysis was determined, after an appropriate amount of β-glucosidase was assimilated to the reactions under optimal pH and temperature (pH 7.0 and 40 °C), with various concentrations of glucose as the substrate. When the glucose concentration reached 30%, the conversion rate of gentiooligosaccharides gradually increased with an increase in the concentration of glucose (Table 1). However, when the glucose concentration was higher than 60%, the output of gentiooligosaccharides no longer improved further, and was replaced by a slow reduction, presumably because the reverse hydrolytic activity of β-glucosidase began to be inhibited by free glucose above this concentration. Furthermore, the demand for the enzyme was more than 400 U·g^−1^ of glucose, which is consistent with a previous report that required high enzyme consumption (900 U·g^−1^ of glucose) [6]. This result indicates that the preparation of gentiooligosaccharides by Blg163-mediated reverse hydrolysis may be an inefficient way of transformation using glucose as a single substrate.

**Figure 3:**
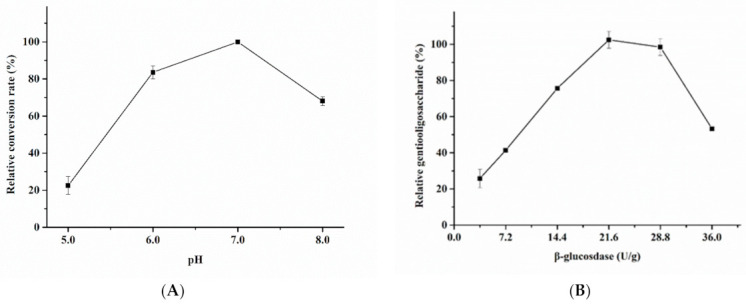
Effects of pH on the yield of gentiooligosaccharides (**A**). The reactions were performed at 40 °C in 0.2 M McIlvaine buffer for pH 3.0–8.0 or 0.05 M glycine-NaOH buffer for pH 8.0–11.0 with 30% glucose and 15% cellobiose. Outputs of gentiooligosaccharides at different concentration of Blg163 (**B**). The reactions were performed in optimum conditions.

β-glucosidase-mediated transglycosylation using a mixed substrate of cellobiose and glucose was adapted for gentiooligosaccharide synthesis. The yield of gentiooligosaccharides first increased and then decreased with an increase in reaction temperature, peaking at 40 °C (Figure 2B). The optimum pH for gentiooligosaccharide production was pH 7.0. More than 70% yield was retained at pH ranging from 6.0 to 8.0 (Figure 3A). As far as we are aware, Blg163 is the first neutral and mesophilic β-glucosidase that exhibits efficient oligosaccharide synthesis activity. This is distinctly different from other β-glucosidases as their optimal temperature and pH for gentiooligosaccharide production were higher than 50 °C and pH 5.0, respectively [6,11,13]. These results indicate that Blg163-mediated gentiooligosaccharide synthesis is simple, cost-effective, and energy-saving without the need for high temperature and sophisticated equipment. All these features highlight the potential application of Blg163 in the food industry for the commercial production of gentiooligosaccharides.

Compared with a single substrate, the mixed substrate of glucose and cellobiose resulted in greater productivity. The peak yield of gentiooligosaccharides was attained at 70.3 g·L^−1^ when the molar ratio of glucose to cellobiose was 4:1 (30% glucose + 15% cellobiose).Steps to increase the proportion of glucose led to a slight rise in the production of gentiooligosaccharides (Table 2). However, the cost price of glucose is higher than that of cellobiose. Thus, the most appropriate molar ratio of glucose to cellobiose can be inferred to be 4:1 to balance yield and cost. Our novel β-glucosidase is different from *Trichoderma viride* β-glucosidase, which attained the highest yield of gentiooligosaccharides with a glucose to cellobiose molar ratio of 1:1 [6].

The optimal β-glucosidase enzyme concentration required for effective yield of gentiooligosaccharides was tested at 40 °C and pH 7.0 with a mixed substrate of glucose and cellobiose (40% glucose + 20% cellobiose) (Figure 2B and Figure 3A). These results indicated that the yield of gentiooligosaccharides first improved with increase in the amount of the enzyme, peaked at 21.6 U/0.5 g glucose/g cellobiose, followed by a gradual reduction. Hence, the optimum dosage of Blg163 was found to be 43.2 U/1.0 g glucose/g cellobiose, as shown in Figure 3B.

**Figure 4:**
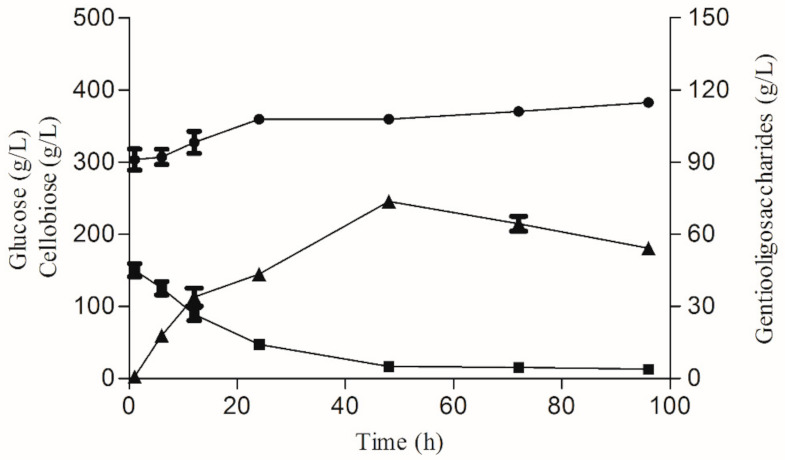
Time course of gentiobiose yield by the recombinant Blg163. The reactions were performed in optimum conditions. The HPLC analysis of the products formed by transglycosylation. (●) Glucose; (■) cellobiose; (▲) gentiooligosaccharides.

The synthesis of gentiooligosaccharides via reverse hydrolytic reactions requires nearly twice the amount of glucose and consumes more than 20-times the amount of β-glucosidase enzyme than transglycosylation. During the transglycosylation process, gentiooligosaccharides and glucose gradually accumulate and cellobiose is gradually deprived until the equilibrium point of the reaction is reached (Figure 4 and Figure S5). The possible reason is that Blg163 has a lower affinity for gentiobiose than cellobiose, so it begins to degrade gentiobiose when the cellobiose is below the threshold, leading to a reduction in gentiooligosaccharides. With increase in reaction time, the yield of gentiooligosaccharides first increased, reached a peak after 48 h, and then decreased gradually. Therefore, we conclude that an optimum time of 48 h was required for completion of the reaction.

## 4. Conclusions

This study identified a novel β-glucosidase gene, *blg163*, from the coral microbial metagenome which was successfully expressed in *Escherichia coli* BL21(DE3) pLysS. Blg163 exhibited a reasonable tolerance to glucose and ethanol, retaining >40% activity in the presence of 5–20% glucose and >50% in the presence of 15% ethanol. Using the reverse hydrolysis activity of Blg163 to synthesize gentiooligosaccharides, we showed that a peak yield of 43.02 ± 3.20 g·L^−1^ and a conversion rate of 5.38 ± 0.40%, with glucose as a single substrate, could be achieved at pH 7.0 and 40 °C. We also synthesized gentiooligosaccharides via β-glucosidase-catalyzed transglycosylation with a mixed substrate of glucose and cellobiose. This process consumed 21.6 U/0.5 g glucose/g cellobiose of β-glucosidase, achieving a yield of 70.34 ± 2.20 g·L^−1^ and a conversion rate of 15.63%. This is the mildest reaction and the lowest β-glucosidase consumption in gentiooligosaccharide synthesis from cellobiose and glucose reported to date.

## 5. Patents

There is a patent (NO. ZL 2020 10343911.X, China) resulting from the work reported in this manuscript.

## Figures and Tables

**Table 1:** Output of gentiooligosaccharides (g·L^−1^) with different concentrations of glucose.

Glucose Concentration	Yields (g·L^−1^)	Conversion Rate (%, *w/w*)
80% glucose	43.02 ± 3.20	5.38 ± 0.40
70% glucose	42.25 ± 4.21	6.07 ± 0.60
60% glucose	40.50 ± 3.20	6.75 ± 1.12
50% glucose	35.34 ± 2.60	7.07 ± 0.52
40% glucose	28.21 ± 2.72	7.05 ± 0.68
30% glucose	17.38 ± 0.78	5.79 ± 0.26
20% glucose	ND	ND
10% glucose	ND	ND

ND, not detected.

**Table 2:** Effect of mass ratio of glucose and cellobiose on gentiooligosaccharide synthesis.

Substrate Concentration	Yields (g·L^−1^)	Conversion Rate (%, *w/w*)
60% glucose + 30% cellobiose	51.21 ± 4.41	5.69 ± 0.49
50% glucose + 25% cellobiose	56.25 ± 3.30	7.50 ± 0.44
40% glucose + 20% cellobiose	63.00 ± 6.70	10.50 ± 1.12
30% glucose + 15% cellobiose	70.34 ± 2.20	15.63 ± 0.49
25% glucose + 12.5% cellobiose	40.39 ± 2.51	10.77 ± 0.66
20% glucose + 10% cellobiose	25.38 ± 0.78	8.46 ± 0.26
10% glucose + 10% cellobiose	9.02 ± 0.42	4.51 ± 0.21
10% glucose + 20% cellobiose	12.84 ± 0.21	4.28 ± 0.07
5% glucose + 20% cellobiose	12.88 ± 0.50	5.15 ± 0.20

## Supplementary Materials

The following are available online at https://www.mdpi.com/article/10.3390/foods10122985/s1, Figure S1: Stabilities of Blg163 under temperature; Figure S2: Stabilities of Blg163 under pH; Figure S3: Stabilities of Blg163 under glucose; Figure S4: Stabilities of Blg163 under ethanol; Figure S5: The gentiobiose production by the purified Blg163, The reactions were performed in 50 mM citrate buffer (pH 5.0) at 40 °C with substrates (15% cellobiose and 30% glucose) and 21.6 U/0.5 g glucose/g cellobiose. 1. glucose; 2. cellobiose; 3. Gentiobiose; 4–8. product of different reaction; Table S1: Substrate specificity and Kinetic parameters of Blg163.
