# Supplementary material for: A Novel Neutral and Mesophilic β-Glucosidase from Coral Microorganisms for Efficient Preparation of Gentiooligosaccharides
Source: Foods. 2021 Dec 3;10(12):2985. doi: 10.3390/foods10122985 (PMC8700683; doi:10.3390/foods10122985)
Supplement: Supplementary file 1 [file foods-10-02985-s001.zip › foods-1461991-supplementary.pdf]

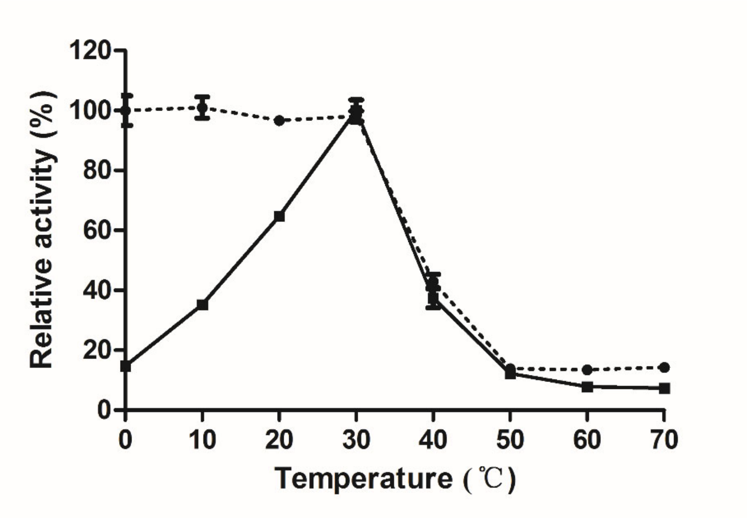

Figure S1. Stabilities of the three types of Blg163 under temperature. All relative activities were calculated by setting to 100% the activity obtained in McIlvaine buffer (pH 7.0) at various temperatures (0 to 40 °C) without glucose and ethanol; Thermostability of Mg132 was assayed after pre-incubation at temperatures 0 to 45 °C for 1 h. The error bars represent the means ± SD (n=3).

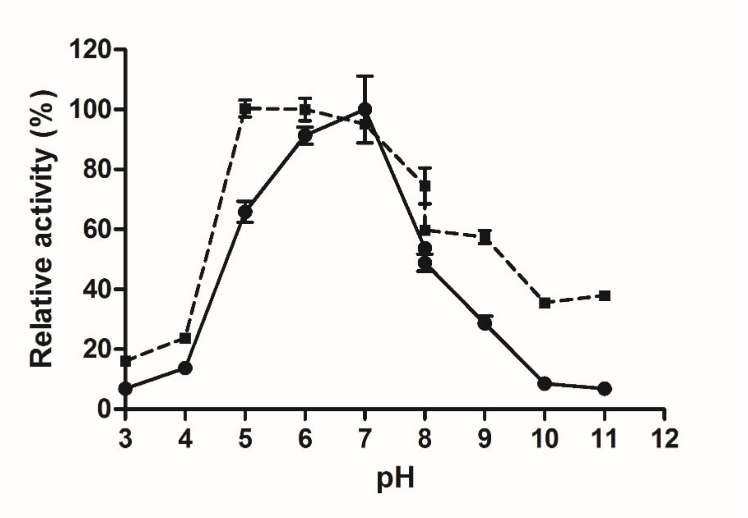

Figure S2. Stabilities of the three types of Blg163 under pH. The stabilities of the three types of BGL were measured using pNPG as the substrate after incubation for 10 min at 35 °C. The error bars represent the means ± SD (n=3).

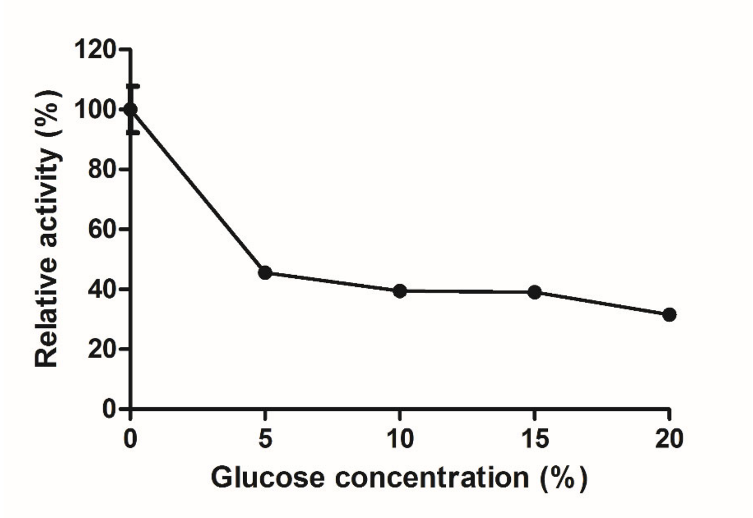

Figure S3. Stabilities of the three types of Blg163 under glucose. The stabilities of the three types of BGL were measured using pNPG as the substrate after incubation for 10 min at 35 °C. All relative activities were calculated by setting to 100% the activity obtained in McIlvaine buffer (pH 7.0) at 35 °C without glucose; The error bars represent the means ± SD (n=3).

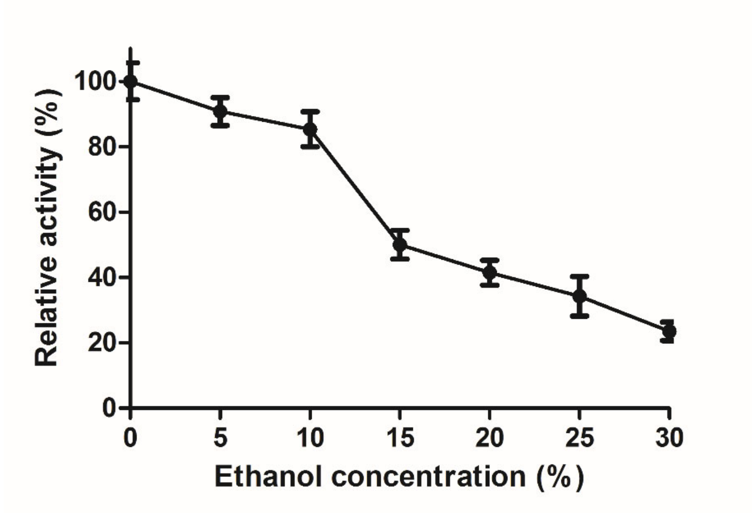

Figure S4. Stabilities of the three types of Blg163 under ethanol. The stabilities of the three types of BGL were measured using pNPG as the substrate after incubation for 10 min at 35 °C. All relative activities were calculated by setting to 100% the activity obtained in McIlvaine buffer (pH 7.0) at 35 °C without ethanol; The error bars represent the means ± SD (n=3).

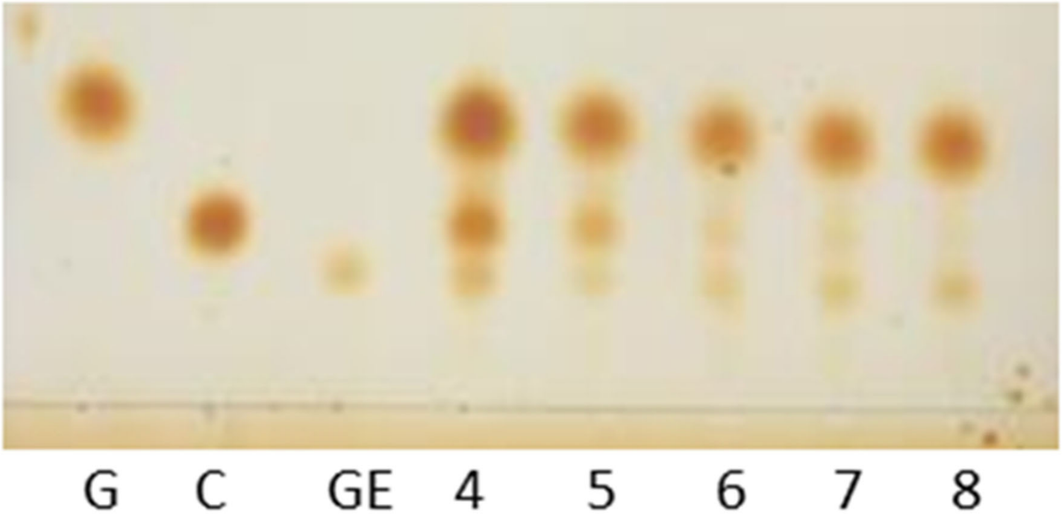

Figure S5. The gentiobiose production by the purified Blg163. The reactions were performed in 50 mM citrate buffer (pH 5.0) at 40 °C with substrates (15% cellobiose and 30% glucose) and 21.6 U/0.5 g glucose/g cellobiose. 1. glucose; 2. cellobiose; 3. Gentiobiose; 4-8. product of different reaction.

Table S1. Substrate specificity and Kinetic parameters of Blg163.

Substrate	Specific activity[ab] ($U \cdot mg^{-1}$)	*km* (mM)
Carboxymethyl cellulose	ND	ND
*p*NPGlu	29.74 ± 2.7	0.32
Cellobiose	28.00 ± 2.3	0.45
Oat spelts xylan	--	0.72

[a]Assay was performed at the optimum condition. [b]Standard deviations were shown behind the specific activities.
